# Hybrid Classification/Regression Approach to QSAR Modeling of Stoichiometric Antiradical Capacity Assays’ Endpoints

**DOI:** 10.3390/molecules27072084

**Published:** 2022-03-24

**Authors:** Petko Alov, Ivanka Tsakovska, Ilza Pajeva

**Affiliations:** 1 Department of QSAR and Molecular Modelling, Institute of Biophysics and Biomedical Engineering, Bulgarian Academy of Sciences, 1113 Sofia, Bulgaria; itsakovska@biomed.bas.bg; 2 Department of Mathematical Modeling and Numerical Analysis, Institute of Mathematics and Informatics, Bulgarian Academy of Sciences, 1113 Sofia, Bulgaria

**Keywords:** antiradical capacity assays, ABTS^●+^, DPPH^●^, TEAC, stoichiometric endpoint, QSAR, classification/regression approach

## Abstract

Quantitative structure–activity relationships (QSAR) are a widely used methodology allowing not only a better understanding of the mechanisms of chemical reactions, including radical scavenging, but also to predict the relevant properties of chemical compounds without their synthesis, isolation and experimental testing. Unlike the QSAR modeling of the kinetic antioxidant assays, modeling of the assays with stoichiometric endpoints depends strongly on the number of hydroxyl groups in the antioxidant molecule, as well as on some integral molecular descriptors characterizing the proportion of OH-groups able to enter and complete the radical scavenging reaction. In this work, we tested the feasibility of a “hybrid” classification/regression approach, consisting of explicit classification of individual OH-groups as involved in radical scavenging reactions, and using further the number of these OH-groups as a descriptor in simple-regression QSAR models of antiradical capacity assays with stoichiometric endpoints. A simple threshold classification based on the sum of trolox-equivalent antiradical capacity values was used, selecting OH-groups with specific radical stability- and reactivity-related electronic parameters or their combination as “active” or “inactive”. We showed that this classification/regression modeling approach provides a substantial improvement of the simple-regression QSAR models over those built on the number of total phenolic OH-groups only, and yields a statistical performance similar to that of the best reported multiple-regression QSARs for antiradical capacity assays with stoichiometric endpoints.

## 1. Introduction

Conversion of the molecular oxygen to reactive oxygen species (ROS) in the process of sequential one-electron reductions occurs constantly in living organisms and could be increased under pathological conditions [1]. A complex defense system has evolved in aerobic organisms for dealing with free radical oxidation. It includes a number of ROS-metabolizing enzymes, metal ion sequestration proteins, and a number of low-molecular-weight compounds that can intercept initiating or chain-carrying free radicals and act as either preventive or chain-breaking antioxidants (α-tocopherol, plant phenols, and polyphenols) [2,3]. The research interest, especially in polyphenolic antioxidants of plant origin, is determined mainly by two factors: (a) dietary polyphenols exert a number of beneficial health effects presumably due to their antioxidant properties; and (b) the enormous number of natural plant phenolic compounds (>8000 identified) provides a vast source of data for experimental and computational research [4,5]. The differentiation of polyphenols by their ability to counteract oxidative processes is not straightforward using classical methods for measuring antioxidant reaction kinetics [6] or relative antioxidant activity [7]. This has led to the development of numerous ways of measuring antiradical capacity [8] widely used for evaluation and differentiation of (poly)phenols’ “antioxidant potency”. Antiradical capacity methods measure the stoichiometry of reactions of (poly)phenols with stable free radicals (e.g., 2,2′-azino-bis(3-ethylbenzothiazoline-6-sulfonic acid), ABTS^●+^, or 2,2-diphenyl-1-picrylhydrazyl, DPPH^●^ [8,9]) or of the reduction of other, non-radical compounds (e.g., Fe^3+^ or Cu^2+^ ions [8,10]).

Structure–activity and quantitative structure–activity relationships (SAR and QSAR) are widely used methodologies allowing not only the better understanding of the mechanisms of pharmacological action [11] and of chemical reactions, including antioxidant and antiradical reactions [12,13,14,15], but also the prediction of the relevant properties of chemical compounds without experimental testing and even without synthesis or isolation in the case of natural compounds. It is not surprising that QSAR approaches have been applied to model and predict the endpoints of many stoichiometric antiradical capacity assays. The simplest approach correlates the number of hydroxyl groups in the (poly)phenol molecule (nOH) with the endpoint of the antiradical capacity assay [16], and indeed, as noted in [10], “among the compounds having the same basic structure, the number of OH groups is the determinant factor for the antioxidant activity”. However, in each particular structure the surroundings of the individual hydroxyl group are of crucial importance for its participation in radical scavenging [10,12,14,17].

The regression QSAR models developed for antiradical capacity assays, in fact, almost always include the number of OH-groups in the (poly)phenol molecule as one of the descriptors in the regression equation [10,18,19], or the number of OH-groups identified as important in the antiradical reactions (e.g., vicinal OH-groups in catechol or pyrogallol moieties) [17,20]. In the models, where nOH is not among the regression parameters, a parameter strongly correlated with nOH is usually present, e.g., the sum of charges of hydroxyl group atoms [21] or cyclic voltammetry peak currents [22]. The rest of the parameters in the QSAR regression models aim to describe the dependence of OH-groups’ reactivity in radical scavenging reactions on their molecular surroundings. These are either indicator variables summarizing the (poly)phenol structural features [19,23,24], cyclic voltammetry-measured oxidation potentials [10,22], or calculated electronic descriptors as the lowest bond dissociation enthalpy (BDE) of the molecular OH-groups [17,18] or spin densities (SD) on hydroxyl radical oxy gen atoms [21].

Notably, among the reported QSARs of antiradical capacity assays, there are no models attempting to use explicit classification of individual hydroxyl groups within a single polyphenol molecule and across multiple tested (poly)phenols with regard to their participation in the radical scavenging reactions [15]. The antiradical capacity assays, due to the stoichiometric nature of their endpoints, could benefit from such a classification. The explicit classification of (poly)phenol OH-groups could be based on easily interpretable calculated electronic parameters, as illustrated by [17,18], where the lowest BDE was used as a descriptor assisting in the classification of simple phenolics or flavonoid compound performance in antiradical capacity assays, but implicitly on the level of whole molecules only. Reasonable candidates for classification descriptors regarding the participation of OH-groups in the radical scavenging are the electronic parameters determining the phenoxyl radical stability, e.g., BDE (predominantly for monophenols [25]) or some spin-densities-related parameters describing spin delocalization on the phenoxyl radicals (predominantly for polyphenols [26,27,28,29]). However, such explicit classification of OH-groups, and its use in QSAR models, has some limitations and requires some important assumptions: (a) it cannot account for some molecular features important in radical stabilization [13,30,31,32] unless they are reflected in the electronic descriptors used; (b) it cannot account for the structural changes in (poly)phenol molecules upon its participation in a number of sequential radical scavenging reactions [33,34]; and thus, (c) it assumes that ranking of the individual OH-groups remains unchanged during the radical scavenging assay, both within a single polyphenol molecule and across the all molecules tested in the assay.

In this work, we tested the feasibility of an approach based on explicit classification of the individual hydroxyl groups in several aspects. First, we showed that an appropriate classification method using calculated electronic radical-stability-related parameters could be derived and reliable regression-based QSAR models could be obtained using the resulting number of “active” OH-groups, i.e., classified as involved in the radical scavenging in the ABTS^●+^ and DPPH^●^ assays. Furthermore, we explored how these “hybrid” classification/regression QSAR models improve upon simple-regression models obtained using the total number of (poly)phenol OH-groups, and addressed the question whether the classification accuracy could be improved by the addition of other, reactivity-related parameters to the classification descriptors. Finally, we checked the applicability domain of the classification/regression QSAR models, i.e., were the calculated classification descriptors consistent across a broad range of structurally diverse (poly)phenols, so that this classification/regression approach could be applied to structurally diverse datasets.

## 2. Data and Methods

### 2.1. Data Selection and Curation

The data from stoichiometric radical scavenging assays were collected from the study of Cai et al. (2006) [16]. The scavenging capacities against stable ABTS^●+^ and DPPH^●^ radicals of 100 phenolic and polyphenolic compounds (predominantly of plant origin) were presented as trolox-equivalent antiradical capacity (TEAC). These compounds belong to the following chemical classes: phenolic acids, chalcones, flavonoids, tannins, coumarins, lignans, quinones, stilbenes, and curcuminoids. They possess between 0 and >15 phenolic groups, and their antiradical capacities range from 0 to >10, in the DPPH^●^ assay, and from 0 to >11, in the ABTS^●+^ assay. In the present study, compounds without phenolic groups (e.g., trans-chalcone, coumarine, anthraquinone) and those with polymeric structures and a variable number of monomers (e.g., Chinese tannin) were excluded (10 compounds altogether). Thus, the compounds in the resulting dataset possessed between 1 and 15 phenolic groups, and their TEAC ranged from 0.020 to 8.79 in the DPPH^●^ assay, and from 0.025 to 9.18 in the ABTS^●+^ assay.

The chemical structures of the selected compounds were retrieved from comprehensive online databases (PubChem [35], ChemSpider [36], NCI/CADD CIR [37]), or built in the CCG Molecular Operating Environment (MOE) [38] if not found in the databases. The chiral centers of the built structures were checked and corrected when necessary. Finally, their InChi keys were used for a reverse check by searching the online databases. All format conversions of the chemical structures’ representations were performed with Open Babel [39].

After the structure retrieval and quality check, another compound (carthamin) was discarded because of a discrepancy between the structure provided in the source [16] and the structures retrieved from online databases [35] and other publications [40,41]. One more compound was excluded, because of the doubtful stability of the myricetin preparation suggested in the source [16]. Thus, the final dataset consisted of 88 curated (poly)phenolic structures with associated ABTS^●+^ and DPPH^●^ radical scavenging data.

### 2.2. Geometry Optimization and Electronic Parameters Calculations

The curated structures were energy minimized using MMFF94x force field in MOE, and then subjected to conformational search using LowModeMD [42] procedure of the Environment (an exhaustive search procedure is not feasible for molecules with saturated and/or fused cycles). The minimal energy conformations were geometry optimized using the semi-empirical molecular orbital package MOPAC2016 [43,44] by AM1, PM6, PM7, and RM1 Hamiltonians, in vacuum or accounting for the solvent contribution to the enthalpies with the conductor-like screening model (COSMO). Semi-empirical methods were used in this study because of their reasonable computational cost and reliability, sufficient for calculation of electronic parameters for modeling of radical scavenging reactions [18].

Cation-radical, oxygen-centered radical, and anion structures were prepared from the basic molecular structures. From the 88 molecular structures, 88 cation radicals, 265 oxygen-centered radicals, and 265 anions were generated. The OH-groups of the sugar moieties in glycosides were not processed; however, some non-phenolic OH-groups were (enol hydroxyls of curcuminoids and chromosomal hydroxyls of flavan-3-ols). The structures were geometry optimized using restricted and unrestricted Hartree-Fock (RHF, UHF) formalisms for non-radical and radical species, respectively. The simulation-terminating criteria were increased hundred-fold using keyword PRECISE, the gradient norm only was explicitly set using keyword GNORM = 0.02. Finally, a single self-consistent field calculation with restricted open-shell Hartree-Fock (ROHF) calculation was performed on the UHF-optimized radical structures to avoid spin contamination, at least at the final stage of enthalpy and spin density calculations. The heats of formation (*H*) and the spin densities on the C- and O-atoms (derived by Löwdin population analysis [45]) were extracted from the MOPAC output files. All simulations were performed on Persy Stinger Intel^®^ Xeon workstations (www.persy.com, last accessed on the 1 December 2021).

The thermodynamic parameters of the different radical scavenging reactions (the hydrogen atom transfer, HAT; the sequential proton loss-electron transfer, SPLET; and the single electron transfer-proton transfer, SET-PT), which are pertinent to the individual OH-groups (the bond dissociation enthalpy, BDE; the proton affinity, PA; the electron transfer enthalpy, ETE; and the proton dissociation enthalpy, PDE) were calculated according to the standard reaction mechanisms. The pertinent to the whole molecule adiabatic ionization potential, IP, was also calculated.

BDE = *H*(PhO^●^) + *H*(H^●^) − *H*(PhOH) (1)

PA = *H*(PhO^−^) + *H*(H^+^) − *H*(PhOH) (2)

ETE = *H*(PhO^●^) + *H*(e^−^) − *H*(PhO^−^) (3)

IP = *H*(PhOH^●+^) + *H*(e^−^) − *H*(PhOH) (4)

PDE = *H*(PhO^●^) + *H*(H^+^) − *H*(PhOH^●+^) (5)

The gas-phase enthalpies of the proton, electron, and hydrogen atom, as well as their solvation enthalpies, were taken from literature [46,47]

### 2.3. Descriptor Calculations and Statistics

The calculation of the parameters necessary for the active/inactive OH-group classification and the descriptor assignment based on this classification, as well as the statistical calculations (simple linear regressions and leave-one-out, LOO, cross-validation procedures) were performed using in-house Perl [48] scripts, employing List::Rank, List::Util, Statistics::Descriptive, Statistics::OLS, and Statistics::Regression modules.

## 3. Results and Discussion

### 3.1. Calculation and Analysis of Electronic Parameters

The classification of the OH-groups of interest (phenolic, enolic in curcuminoids, and chromanol OH-groups of flavan-3-ols) as active or inactive in radical scavenging reactions was based on the calculated electronic parameters determining radical stability. The BDE and the spin delocalization were used for this purpose [25,26,27,28,29]. Having in mind the substantial structural diversity of the dataset used, we chose the maximal SD localized on any of the heavy atoms of oxygen-centered radicals (maxSD) as a measure of the spin delocalization on radical structures (lower maxSD indicates more spin delocalization) instead of using SD sums/normalized sums over the oxygen or aromatic atoms in the compounds’ radicals [27,28,29,49].

BDE and maxSD were obtained from semi-empirical simulations of all 88 mono- and polyphenolic compounds under investigation. Compound structures and calculated electronic parameters are reported in the Supplementary SDF files.

**Figure 1:**
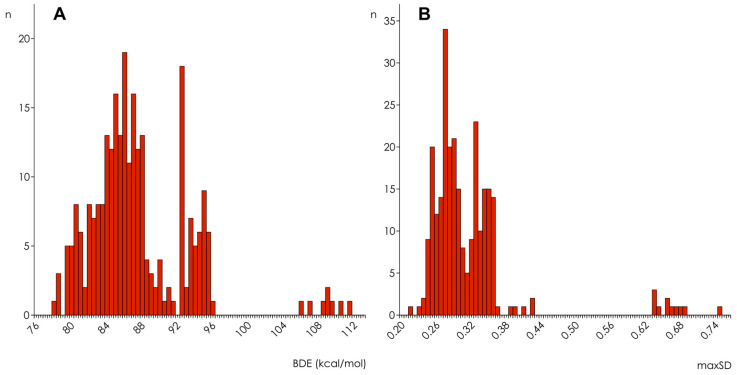
Distribution histograms for BDE (**A**) and maxSD (**B**) obtained in semi-empirical simulations using AM1 Hamiltonian without COSMO corrections (*n* = 265).

The distribution of BDE and maxSD over all compounds’ OH-groups of interest calculated by AM1 are shown in Figure 1. Three different distribution modes can be outlined, and this trimodal pattern was characteristic for the results obtained in simulations with all other Hamiltonians too, with or without COSMO corrections. The analysis of OH-groups belonging to the three distribution modes revealed some differences between BDE and maxSD distributions. In the case of BDE, the first mode (78 ÷ 92 kcal/mol) contains mainly OH-groups in ortho-position one to another (e.g., those of caffeic acid, gallic acid, catechin, and quercetin OH-groups in the flavonoid B-ring—for flavonoid ring labeling see [12]), to a carbonyl group (e.g., 3-OH groups of flavonols), or to a methoxy group (e.g., hesperetin OH-group in the B-ring). In the second mode (92 ÷ 96 kcal/mol) OH-groups in meta- or para-position one to another (e.g., chrysin and kaemferol OH-groups in the A-ring, or those of 1,5-dihydroxylanthraquinone) and OH-groups without a counterpart in the benzene ring (e.g., those of o-hydroxybenzoic acid and rhein), were mainly found. The third mode (BDE > 96 kcal/mol) consisted of 3-OH groups of flavanols and condensed tannins. MaxSD distributions differed from those of BDE mainly by the relocation of the 3-OH groups of flavonols from the lowest end of the first mode into the highest end of the second mode (0.30 ÷ 0.42) and by relocation of the enol hydroxyl groups of curcuminoids from the first mode into the third mode (maxSD > 0.42).

Due to the described pattern of differences in BDE and maxSD distributions and to the presence of both mono- and polyphenols in the dataset, a composite descriptor was constructed, BDE × maxSD, in order to encompass the stability measures of both compound types and to account for differences in the primary parameters’ distributions. The multiplication product of the primary descriptors was chosen instead of their sum to avoid predicaments caused by the different scales of BDE and maxSD (medians of BDE varying between 79.2 and 86.2 for different Hamiltonians and solvation models, and between 0.279 and 0.320 in case of maxSD).

### 3.2. Hydroxyl Group Classification and Regression Model Construction

**Figure 2:**
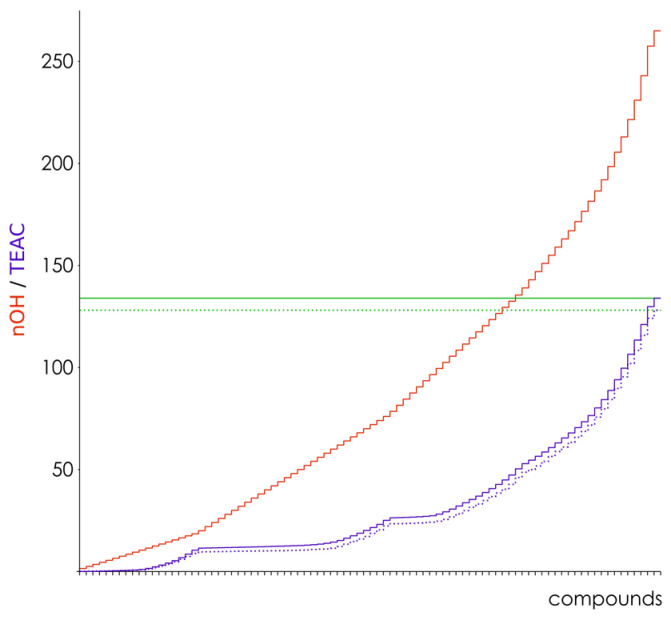
Cumulative graph of the hydroxyl groups (red) and of TEAC values (purple) over all compounds in the ABTS^●+^ (solid line) and DPPH^●^ (dotted line) datasets. The thresholds defined are shown in green lines. For graph clarity, compounds are sorted by the number of OH-groups and by antiradical capacity, and their names are omitted on the graph (the names and numerical data are provided in Supplementary Table S1).

For hydroxyl group classification as involved or not in radical scavenging (referenced further as “active” or “inactive”), we chose the simplest and the most easily interpretable method—a threshold classification. Bearing in mind that TEAC values are roughly phenolic equivalents, we assumed that the TEAC sum over all tested compounds represented the number of active OH-groups in the dataset and the rest of the OH-groups, up to their total number in the dataset, were inactive. Thus, the threshold value of the electronic parameters could be defined (Figure 2). Total OH-groups of interest were 265, and the TEAC sums were 133.98 and 128.08 for ABTS^●+^ and DPPH^●^, respectively. Accordingly, we assumed to have 134 active vs. 131 inactive OH-groups in the ABTS^●+^ dataset, and 129 active vs. 136 inactive OH-groups in the DPPH^●^ dataset. Thus, the values corresponding to the 134th (ABTS^●+^) or 129th (DPPH^●^) sorted parameters were used as thresholds for distinguishing between active or inactive OH-groups in each compound in the datasets. Any OH-group with a parameter value less or equal to the threshold was classified as active, and vice versa. The number of active OH-groups in each molecule was then used as an independent variable in a simple linear regression with the TEAC value as a dependent variable.

Having quite large datasets, we were in a position to check how the calculated thresholds behave with increasing the number of compounds used for their calculation. Our assumption was that by increasing the number of compounds used for the threshold calculation, the threshold values should converge to the final value obtained with all compounds. Otherwise, the final threshold value would be random and of no significant quality to perform any reasonable classification.

**Figure 3:**
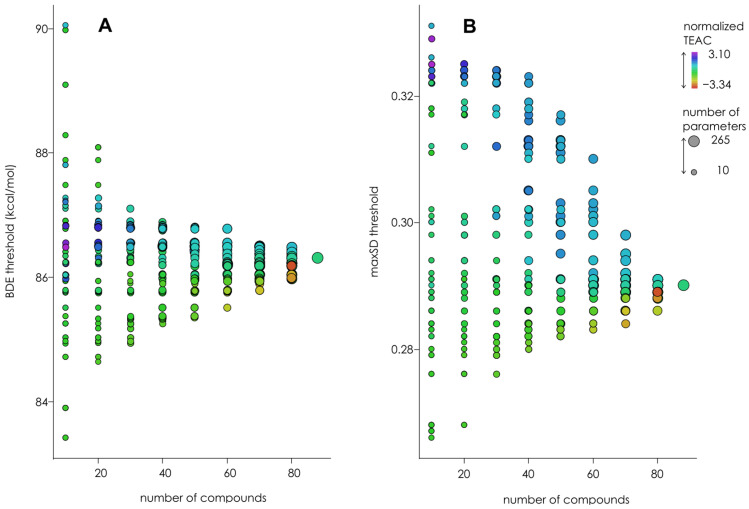
Convergence of BDE (**A**) and maxSD (**B**) thresholds calculated by “sliding” approach using ABTS^●+^ experimental data. The electronic parameters were obtained in AM1 simulation without COSMO corrections. Color codes represent the TEAC sum for each subgroup, normalized over the subgroups with the same number of compounds. The size of the points represents the number of parameters falling into any given subgroup.

**Figure 4:**
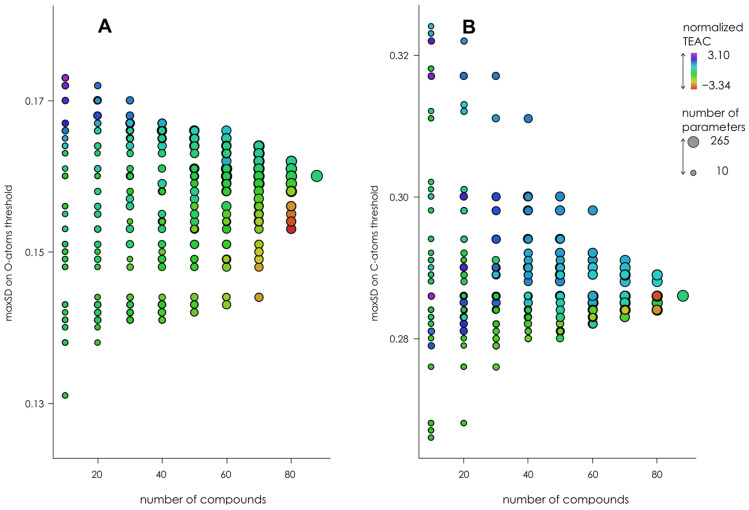
Convergence of maxSD_O_ (**A**) and maxSD_C_ (**B**) thresholds calculated by “sliding” approach using ABTS^●+^ experimental data. The electronic parameters were obtained in AM1 simulation without COSMO corrections. Color codes represent the TEAC sum for each subgroup, normalized over the subgroups with the same number of compounds. The size of the points represents the number of parameters falling into any given subgroup.

The convergence test was performed using “sliding” approach—subgroups of compounds were formed consisting of 10, 20, 30 … 80 compounds each and starting with the 1st, 2nd, 3rd … 88th compound, and thresholds were calculated for each subgroup. During the subgroups’ formation, the compounds were sorted by the OH-groups’ number and antiradical capacity; thus, each group contained a very different number of OH-groups. The results of these experiments are shown in Figure 3. It can be seen that while BDE thresholds converge decently, maxSD convergence is somewhat unsatisfactory. Thus, we extracted separately maxSD on C- and O-atoms and checked the convergence of classification thresholds calculated from maxSD_O_ and maxSD_C_. The results presented in Figure 4 clearly show that the poor maxSD_O_ thresholds convergence is responsible for unsatisfactory convergence of “unseparated” maxSD thresholds, while maxSD_C_ thresholds converge considerably better. Thus, in the further modeling experiments, we used maxSD_C_ and BDE × maxSD_C_ as classification parameters. The threshold values obtained for BDE, maxSD_C_, and BDE × maxSD_C_ are shown in Table 1.

**Figure 5:**
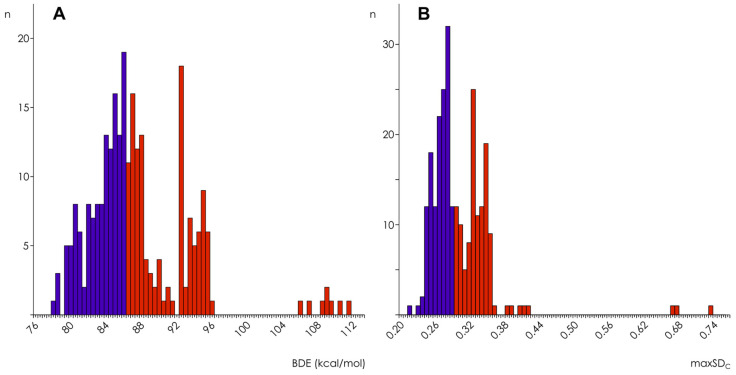
Distribution histograms of active and inactive hydroxyl groups by BDE (**A**) and maxSD_C_ (**B**) threshold classifications in semi-empirical simulations using AM1 Hamiltonian without COSMO corrections (*n* = 265). Red—inactive groups, purple—active groups.

Applying these thresholds, we split the OH-groups into classes, putatively active or inactive in antiradical capacity assays (Figure 5). In order to check the classification accuracy, we built ordinary linear regression models with the number of active groups in the tested molecules as an independent variable and compared their squared correlation coefficients r^2^ and slopes to those of the linear regression model using the total number of phenolic hydroxyl groups as an independent variable (Supplementary Table S1). The correlation coefficients, which reflect goodness-of-fit of the entire model, increased slightly: in the case of the ABTS^●+^ assay from 0.817 for total OH-groups model to 0.856, 0.836, and 0.832 for BDE, maxSD_C_, and BDE × maxSD_C_ classifications, respectively, and in the case of DPPH^●^ assay from 0.807 for total OH-groups model to 0.827, 0.831, and 0.823 for BDE, maxSD_C_, and BDE × maxSD_C_ classifications, respectively. The slopes, which reflect the classification accuracy for each individual structure, were increased more than the correlation coefficients, approaching 1.0: in the case of the ABTS^●+^ assay from 0.692 for total OH-groups model to 0.940, 0.894, and 0.877 for BDE, maxSD_C_, and BDE × maxSD_C_ classifications, respectively, and in the case of the DPPH^●^ assay from 0.677 for total OH-groups model to 0.936, 0.877, and 0.858 for BDE, maxSD_C_, and BDE × maxSD_C_ classifications, respectively. These observations clearly indicated that classification of the individual OH-groups based on electronic parameters pertinent to them increased the accuracy and prediction quality of the regression models of antiradical assays studied. However, none of the models based on active group numbers has r^2^ exceeding 0.9, while some multiple linear regression (MLR) models based on OH-group numbers (total or vicinal) plus BDE [17,18,20,32] or on EVA vector descriptors [50] are reported to possess r^2^ higher than 0.9, thus suggesting the necessity for further optimization of the proposed models.

### 3.3. Optimization of the Classification/Regression Models

**Figure 6:**
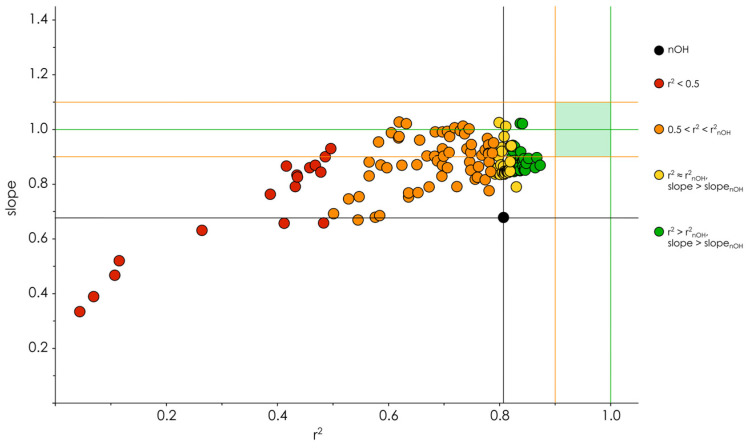
Plot of linear regression models’ slopes vs. squared correlation coefficients r^2^ colored by their accuracy grouping. Black lines correspond to the r^2^ and slope of the “unclassified” nOH model (0.807 and 0.677, respectively). Green lines denote r^2^ = 1 and slope = 1, orange lines—r^2^ = 0.9 and slope = 0.9 and 1.1. Pale green rectangle outlines the desirable zone for the models’ parameters. The plotted models were built using DPPH^●^ assay data.

To explore further the plausibility of the OH-groups classification approach, we tested more classification parameters—alone and in combination. These were reactivity-related electronic parameters, often used to describe the kinetic aspects of radical scavenging reactions [9,51,52]—PA, ETE, IP, and PDE, and their multiplication products with BDE, maxSD_C_, and BDE × maxSD_C_. For IP, only combined classification parameters were used, since being an integral molecular descriptor, it can contribute to intermolecular classifications rather than to the intramolecular ones. In the case of parameters, exhibiting also negative values (PDE in simulations with COSMO corrections), they were offset by a fixed amount (30 kcal/mol) to render all values positive. Such an offset did not affect threshold classifications but was necessary for the construction of correct composite descriptors. The threshold convergence was assessed for the reactivity-related descriptors and their combinations with radical-stability-related parameters, and the convergences were satisfactory for all of them. Two collections of simple-regression models were built (149 models per assay, including those based on nOH_total_) and their correlation coefficients and slopes were analyzed (Figure 6).

Obviously, faulty classifications were present in the collections (colored in red and orange in Figure 6), which was expected—since most antiradical capacity assays, including ABTS^●+^ and DPPH^●^, are designed to achieve the reaction completion [8,53], it is not reasonable to expect a sensible classification using reactivity-related parameters. The opposite results should be quite suspicious and could mean that any decrease (uniform or not) of the independent variable figures may increase the regression accuracy.

Furthermore, the classification/regression models with internal predictive ability (assessed by the LOO, cross-validation correlation coefficients, q^2^_LOO_) higher than that of the models based on the total OH-group number, were analyzed with respect to the nature of their classification parameters and to the simulation conditions used for their calculation. In the two model collections (296 models, excluding those based on nOH_total_), there were 105 models (35%) with q^2^_LOO_ better than those of nOH_total_-based models (49 for the ABTS^●+^ assay and 56 for the DPPH^●^ assay). Among them, only two models were based on the classification with the reactivity-related descriptor (ETE), which underlined the expected leading contribution of phenoxyl radical-stability-related parameters in the classification of the OH-groups for subsequent regression modeling of antiradical capacity assays. Of the remaining 103 models, 31 were obtained by classifications based only on radical-stability-related parameters, and 72 were obtained by classifications based on combined descriptors, including both radical-stability- and reactivity-related parameters. This suggests that accounting for the (poly)phenols reactivity is also of significant importance in classification/regression modeling of antiradical capacity assays.

No substantial differences were observed between the different simulation conditions used for classification parameter calculation: 42% of the highly predictive models were obtained using parameters calculated with COSMO solvation corrections, 58%—without, 28% and 24% of the highly predictive models were obtained using parameters calculated with AM1 and RM1 Hamiltonians, 20% and 29%—with PM6 and PM7 Hamiltonians, respectively.

Inspection of the best ten models for each assay (with the highest q^2^_LOO_ and slopes closest to 1.0) showed that 20% of them were based on BDE-related classification, another 20% on maxSD_C_-related classification, and 60% on BDE × maxSD_C_-related classification in both assays. Only one of these models was based on classification by combined radical-stability-related descriptors alone (BDE × maxSD_C_ in case of DPPH^●^ assay), all other classification descriptors also included reactivity-related parameters (60% PA, 10% ETE, and 25% IP). The dominance of BDE × maxSD_C_-related and PA/ETE-reactivity-related parameters in the classification descriptors among the models with the best accuracy is not surprising—BDE × maxSD_C_ reflects the radical stability of both mono- and polyphenols [25,26,27,28,29] and PA and ETE are relevant to the SPLET reaction, which is assumed operative in the ABTS^●+^ and DPPH^●^ assays [51,54,55].

In contrast to the even distribution of COSMO- and vacuum-calculated classification parameters in the collection of 105 highly predictive models, 85% of the best models used classification parameters obtained in simulations without COSMO solvent corrections. Distribution of the classification parameters obtained in AM1/RM1 or in PM6/PM7 simulations followed the one in the collection of 105 highly predictive models—they were split almost equally (11 and 9, respectively).

The models with the highest q^2^_LOO_ (Table 2) were based on BDE × PA classification for the ABTS^●+^ assay and on BDE × maxSD_C_ × PA classification for the DPPH^●^ assay. The q^2^_LOO_ values of these models were higher than those of nOH_total_-based models by about 0.07, and their slopes were closer to 1.0 compared to the slopes of nOH_total_-based models by about 0.18.

Up to this point we confirmed our hypothesis that building simple-regression models of antiradical capacity of (poly)phenols based on the number of “active” OH-groups, as classified by the electronic parameters pertinent to individual groups, could provide a better statistical quality compared to models built with the total number of OH-groups in the (poly)phenolic molecules. We analyzed the stability descriptors used for OH-group classification and demonstrated that spin densities over carbon atoms only should be used for this purpose. We showed that combining stability with reactivity descriptors improved the classification accuracy in general, but we were not able to obtain classification/regression models with the statistical quality of the best reported MLR models. It should be noted, however, that the datasets used in the reported MLR modeling bore much less structural diversity than the dataset used in the present study—they consisted exclusively of flavonoids [18,19] or simple phenolics [17,32]. Thus, our further modeling experiments considered splitting of the used dataset in two, less chemically diverse parts.

### 3.4. Testing of Classification/Regression Modeling on Less Chemically Diverse Datasets

As described above, our dataset, extracted from Cai et al., 2006 [16], consisted of hydroxycinnamic and hydroxybenzoic acids, flavanols, flavonols, chalcones, flavones, flavanones, isoflavones, condensed tannins, stilbenes, curcuminoids, coumarins, furocoumarins, lignans, anthra- and naphthoquinones. We were reluctant to use the standard selection of flavonoids, since some of them were more similar to non-flavonoid compounds than to the rest of flavonoids (e.g., chalcones to stilbenes and lignans), and vice versa, some non-flavonoid compounds bore significant similarity to flavones (condensed tannins and coumarins). Thus, we split the dataset based on presence of chroman/chromene/chromanon/chromanol [56] moiety in the (poly)phenol structure. The “chromans” subgroup consisted of flavanols, flavonols, flavones, flavanones, isoflavones, condensed tannins, coumarins, and furocoumarins (40 compounds). The “non-chromans” subgroup consisted of hydroxycinnamic and hydroxybenzoic acids, chalcones, stilbenes, curcuminoids, lignans, and anthra- and naphthoquinones (48 compounds). Information for individual compounds participance in each of the groups is provided in Supplementary Tables S2 and S3 and the SDF files.

**Figure 7:**
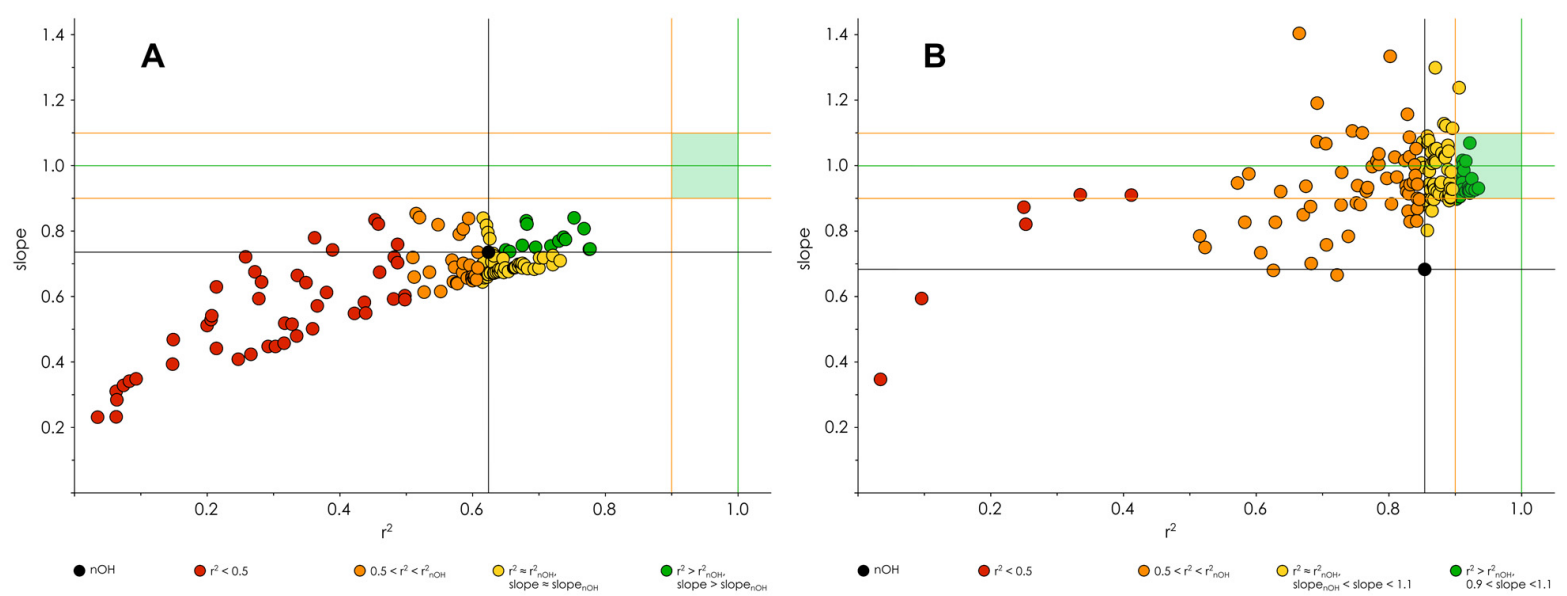
Plot of linear regression models’ slopes vs. squared correlation coefficients colored by their accuracy grouping for non-chromans (**A**) and chromans (**B**) groups of compounds. Black lines correspond to the r^2^ and slope of the “unclassified” nOH models (0.624 and 0.736, respectively, for non-chromans; 0.854 and 0.684, respectively, for chromans). Green lines denote r^2^ = 1 and slope = 1, orange lines—r^2^ = 0.9, slope = 0.9 and 1.1. Pale green rectangle outlines the desirable zone for the models’ parameters. The plotted models were built using DPPH^●^ assay data. Note the differences in color coding between panels (**A**) and (**B**).

For each of the two structural subgroups the procedures described in 0 and 0 were repeated and the results are shown in Figure 7 and provided in Supplementary Tables S2 and S3. As illustrated in Figure 7, the separation of classifications of OH-groups into faulty and satisfactory, observed for the complete dataset, was preserved in both subgroups.

In the non-chromans group, there were 93 models (31%) with q^2^_LOO_ better than those of nOH_total_-based models (36 for the ABTS^●+^ assay and 57 for the DPPH^●^ assay). Among them, only 5 models were based on classification with a reactivity-related descriptor alone (3 on ETE, 2 on PA), 24 models were built on classifications based on radical-stability-related parameters alone, and 64 were built on classifications based on combined descriptors, including both radical stability- and reactivity-related parameters, which is in consonance with the results obtained for the complete dataset.

Distribution of the simulation conditions used for calculation of the classification parameters was similar to that observed in the complete dataset: 40% of the highly predictive models were obtained using parameters calculated with COSMO solvation corrections, 60%—without, 22% and 18% of the highly predictive models were obtained using parameters calculated with AM1 and RM1 Hamiltonians, 26% and 34%—with PM6 and PM7 Hamiltonians, respectively.

Inspection of the best ten models for each assay (with the highest q^2^_LOO_ and slopes closest to 1.0) showed that 55% of them were based on pure BDE classification, 30%—on BDE × PA, and 15%—on BDE × ETE classifications. Notably, none of the models with the best accuracy in the non-chromans subgroup was built on maxSD_C_-related classification. This could be explained by the higher proportion of monophenols and inactive diphenols in this subgroup as compared to the complete dataset (58% vs. 45%), and correspondingly, the higher weight of BDE in determination of the radical stability [25,26]. Exclusive presence of PA and ETE reactivity-related parameters in combined classification descriptors in this group of models is in accordance with SPLET mechanism dominating in the ABTS^●+^ and DPPH^●^ assays [51,54,55].

The distribution of COSMO- and vacuum-calculated classification parameters used in the best 20 models was similar to that in the collection of 93 highly predictive models (35% vs. 65%, respectively). Distribution of classification parameters obtained in AM1/RM1 or in PM6/PM7 simulations was also similar to that in the collection of the highly predictive models (30% vs. 70%, respectively).

The models with the highest q^2^_LOO_ (Table 3) were based on BDE × PA classification for both ABTS^●+^ and DPPH^●^ assays. The q^2^_LOO_ of these models were higher than those of nOH_total_-based models by about 0.22, their slopes were not substantially closer to 1.0 compared to the slopes of nOH_total_-based models—the difference was less than 0.01.

In the chromans group there were 142 models (48%) with q^2^_LOO_ higher than those of nOH_total_-based models (66 for the ABTS^●+^ assay and 76 for the DPPH^●^ assay)—a proportion substantially higher than in either non-chromans group, or in the complete dataset. Among them, only 4 models were based on classification with a reactivity-related descriptor alone (ETE), 37 models were built on classifications based only on radical-stability-related parameters alone, and 101 were built on classifications based on combined descriptors, including both radical stability- and reactivity-related parameters, which is similar to the results obtained for the non-chromans group and the complete dataset.

Again, no substantial differences were observed in the simulation conditions used for classification parameter calculation: 47% of the highly predictive models were obtained using parameters calculated with COSMO solvation corrections, 53%—without; 26% and 23% of the highly predictive models were obtained using parameters calculated with AM1 and RM1 Hamiltonians, 21% and 30%—with PM6 and PM7 Hamiltonians, respectively. In general, these proportions did not differ much from those observed in the complete dataset and in the non-chromans group.

Inspection of the best ten models for each assay (with the highest q^2^_LOO_ and slopes closest to 1.0) showed that none of them were based on BDE-related classification, 40% were based on maxSD_C_-related classification, and 60% on BDE × maxSD_C_-related classification. Thirty-five percent of these models were based on classification by radical-stability-related descriptors alone (maxSD_C_ or BDE × maxSD_C_), the remaining 65% of the classification descriptors also included reactivity-related parameters (25% PA, 5% ETE, 25% IP, and 10% PDE). Distribution of SPLET- and SET-PT-related parameters, unlike those in the non-chromans group and in the complete dataset, was almost even, which does not allow linking their contribution to the mechanisms of radical scavenging reactions in the ABTS^●+^ and DPPH^●^ assays. The lack of pure BDE-related classification descriptors could be explained by the lower proportion of monophenols and inactive diphenols in this subgroup as compared to the complete dataset and the non-chromans (30% vs. 45% and 58%), and correspondingly, the higher weight of spin density vs. BDE in determination of the radical stability [26].

The distribution of COSMO- and vacuum-calculated classification parameters used in the best 20 models was similar to that in the collection of 142 highly predictive models (45% vs. 55%, respectively). The distribution of the classification parameters obtained in AM1/RM1 or in PM6/PM7 simulations, however, differed significantly from that in the collection of the highly predictive models (75% vs. 25%, respectively).

The models with the highest q^2^_LOO_ (Table 4) for both ABTS^●+^ and DPPH^●^ assays were based on maxSD_C_ × IP classification. Since the IP is assumed to be the most fundamental reactivity descriptor defining phenols’ reactivity [57,58,59], its presence in the most successful classification parameters identified in this study is reasonable. The q^2^_LOO_ of these models were higher than those of nOH_total_-based models by about 0.10, their slopes were closer to 1.0 compared to the slopes of nOH_total_-based models by about 0.25. The magnitudes of improvement of correlation coefficients and slopes for the most accurate classification/regression models in the chromans subgroup were about 1.5 times higher than the magnitudes observed in the complete dataset, and the accuracy of these models reached the desirable zone of r^2^ and q^2^_LOO_ higher than 0.9 and slopes between 0.9 and 1.1.

**Figure 8:**
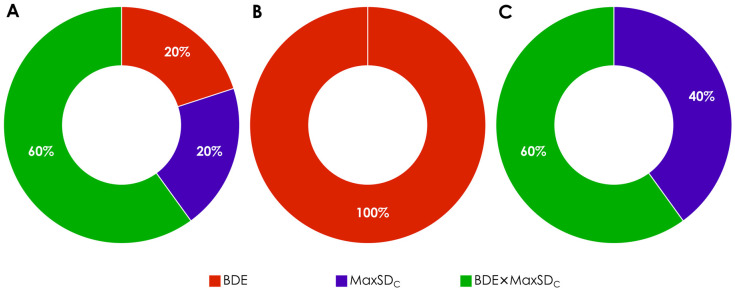
Distribution of radical-stability-related electronic parameters in the descriptors with the highest classification performance (best 20 models, 10 for each of the ABTS^●+^ and DPPH^●^ assays) for the explored datasets. (**A**)—original dataset (88 compounds), (**B**)—non-chromans subgroup (48 compounds), (**C**)—chromans subgroup (40 compounds).

There were distinctive differences between the most accurate classification/regression models of the two compound subgroups with less chemical diversity than the original dataset. First, radical-stability-related descriptors in the combined descriptors with the highest classification performance did not overlap between the non-chromans and chromans subgroups (Figure 8). All best classifying descriptors for the non-chromans group include BDE, and none of them maxSD_C_, while the opposite is true for the chromans group—all best classifying descriptors included maxSD_C_, and 40% of them do not include BDE. Next, the best classifying descriptors in the non-chromans group were obtained in simulations without solvent corrections, while those in the chromans group were obtained in simulations with COSMO corrections. Finally, there were distinct patterns of models’ accuracy improvement for each of the subgroups—while the correlation coefficients improved more for the non-chromans models than for the chromans models (3 vs. 1.5 times when compared to the nOH_total_-based models), the slopes of the non-chromans models did not improve substantially in comparison to the nOH_total_-based models (Figure 7).

The first difference could be explained with the higher proportion of monophenols in the non-chromans subgroup and the different electronic parameters describing the radical stability in mono- and polyphenols, as already commented earlier in this section. It has, however, an implication on the possibility of building “universal” classification/regression models explaining the antiradical capacity of a broad range of structurally diverse phenols—if the optimal classifying descriptors differ for mono- and polyphenols, it would not be possible to build “universal” models with broad applicability domains.

As of the second difference, it could be related to the previous one, in the stability-related classification descriptors for the chromans and non-chromans subgroups. The best models’ classification descriptors containing BDE were obtained in simulations without solvent corrections (in the non-chromans group), while those containing maxSD_C_ were obtained in simulations with COSMO corrections (in the chromans group). Neither in our data, nor in the available literature did we find an explanation of this difference; thus, one can only speculate that electronic-energy-dependent parameters (e.g., BDE) are less sensitive to solvent corrections than charge-dependent ones (e.g., maxSD_C_).

Considering the third difference, the one in the models’ improvement patterns between non-chromans and chromans subgroups, we presumed that besides the structural similarity there should be some other factor(s) determining the changes in the accuracy of classification/regression models of antiradical capacity of (poly)phenols during the refinement procedures. One such factor could be the differences in descriptor and response ranges between the two subgroups of (poly)phenolic compounds. The non-chromans subgroup consisted almost exclusively of compounds possessing 1 to 4 hydroxyl groups (98%), and monophenols and diphenols accounted for more than half of the subgroup. In contrary, compounds possessing more than 4 hydroxyl groups (up to 15 in procyanidin C1) accounted for 28% of the chromans subgroup, while monophenols and diphenols accounted for about one third of the subgroup. A similar situation was observed for the TEAC in both assays—in the non-chromans subgroup they maxed at 3.52 (ABTS^●+^) and 3.92 (DPPH^●^), with a single exception of corilagin (7.76 and 6.98 for ABTS^●+^ and DPPH^●^, respectively). In the chromans subgroup the percentage of TEAC values exceeding 3.5 in each assay was 20% of the data. Therefore, for the non-chromans subgroup, the narrow descriptor and response ranges inevitably exaggerate the noise contribution to the statistical modeling procedures, thus explaining the worse accuracy of the models in this subgroup.

Up to this point, neither of our models based on the number of “active” hydroxyl groups classified as such by the electronic parameters pertinent to the individual OH-groups has a broad applicability domain. Moreover, the differences in best classifying parameters for subgroups dominated by different phenolic populations (mono- vs. polyphenols) do not support expectations to obtain “universal” classification/regression models, able to explain the antiradical capacity of a broad range of structurally diverse phenols. However, building models over a less chemically diverse dataset containing only chroman moiety-possessing compounds, we were able to obtain classification/regression models with the statistical quality of the best reported MLR models (q^2^_LOO_ > 0.9), indicating good classification accuracy over this subset of (poly)phenolic molecules. The models’ slopes and Y-axis intercepts were closer to one and zero, respectively, than those of unclassified models, thus indicating good classification accuracy over the hydroxyl groups of the single polyphenolic molecules. The differences in the models’ improvement patterns suggest that domination of the original dataset by compounds with a small number of hydroxyl groups and small TEAC exaggerates the contribution of the noise to the statistical procedures, thus lowering the classification accuracy of the “universal” models.

## 4. Conclusions

In the present work we showed that a classification based on easily interpretable descriptors, i.e., calculated electronic properties pertinent to the individual hydroxyl groups, could provide an accurate estimation of the number of OH-groups able to scavenge organic radicals ABTS^●+^ and DPPH^●^ in widely used antiradical capacity assays, despite the limitations and assumptions of the approach detailed in the Introduction. We identified a characteristic spin density parameter, namely the maximal spin density on carbon atoms of the phenoxyl radical, as a reliable radical stability descriptor.

We showed that combining two radical stability descriptors, BDE and maxSD_C_, provides more accurate classification of the hydroxyl groups as “active” than that using only a single stability descriptor in the group of structurally diverse phenols. In order to increase the classification accuracy, we combined stability-related descriptors with reactivity-related ones and ascertained that combined stability/reactivity descriptors (e.g., maxSD_C_ × IP) provide even more accurate classification than a combination of stability descriptors only. Refinement of classification/regression models over less chemically diverse phenols showed that the accurate classification of the OH-groups is achieved by different radical stability descriptors in monophenols (BDE) and polyphenols (maxSD_C_), confirming conclusions about the drawbacks of estimation of radical-stabilization enthalpies by BDE alone [26]. The accurate classification of OH-groups resulted in the building of highly predictive simple-regression models of the endpoints from ABTS^●+^ and DPPH^●^ antiradical capacity assays, with q^2^_LOO_ values approaching 0.95.

Having extensive published data only available on antiradical capacity assays performed in polar media, we could not evaluate the full significance of the solvation models in simulations used to calculate classification descriptors. However, decent results obtained with descriptors calculated in vacuum provide encouragement that this classification approach could be successfully used with assays performed in non-polar media. The level of theory used in this study (semi-empirical calculations in molecular orbital package MOPAC2016) provides reliable classifications combined with a low computational cost, confirming conclusions of previously published MLR modeling results of antiradical capacity using the same computational methods [18,29]. Pilot experiments with DFT level of theory calculations of classification descriptors provide indications that classification accuracy could be improved, but it is questionable whether the computational cost of DFT methods is reasonable for QSAR modeling.

In general, our models can be useful tools for the estimation of antiradical activity of various (poly)phenols, including non-tested and virtual ones, and could meet the demand for further and more profound estimation of antioxidant properties of biologically active compounds from this chemical class.

## Figures and Tables

**Table 1:** Thresholds for distinguishing between active and inactive hydroxyl groups for BDE, maxSD_C_, and BDE × maxSD_C_ classification parameters obtained in different semi-empirical simulations for ABTS^●+^ and DPPH^●^ assays.

Hamiltonian	Parameter	Thresholds			
		Vacuum		COSMO	
		ABTS^●+^	DPPH^●^	ABTS^●+^	DPPH^●^
AM1	BDE	86.2	86.0	82.6	82.0
	maxSD_C_	0.286	0.283	0.278	0.274
	BDE × maxSD_C_	25.5	25.2	23.8	22.7
PM6	BDE	84.8	84.3	80.8	80.2
	maxSD_C_	0.315	0.313	0.303	0.300
	BDE × maxSD_C_	28.0	27.5	24.5	24.0
PM7	BDE	85.8	85.2	82.4	82.0
	maxSD_C_	0.296	0.294	0.298	0.292
	BDE × maxSD_C_	27.1	26.5	25.1	24.5
RM1	BDE	83.8	83.5	79.2	79.1
	maxSD_C_	0.299	0.296	0.291	0.290
	BDE × maxSD_C_	25.7	25.7	24.3	24.1

**Table 2:** Models with the highest internal predictive ability obtained for ABTS^●+^ and DPPH^●^ assays, compared to those obtained without OH-group classification. For q^2^_LOO_ calculations, all thresholds were recalculated for each LOO model.

Assay	Simulation Type	Classification Parameter	Model (*n* = 88)
ABTS^●+^	PM6, vacuum	BDE × PA	TEAC = 0.206 + 0.871 × nOH_active_ r^2^ = 0.887, q^2^_LOO_ = 0.875
	–	–	TEAC = −0.562 + 0.692 × nOH_total_ r^2^ = 0.817, q^2^_LOO_ = 0.802
DPPH^●^	RM1, vacuum	BDE × maxSD_C_ × PA	TEAC = 0.206 + 0.859 × nOH_active_ r^2^ = 0.864, q^2^_LOO_ = 0.860
	–	–	TEAC = −0.582 + 0.677 × nOH_total_ r^2^ = 0.807, q^2^_LOO_ = 0.792

**Table 3:** Models with the highest internal predictive ability obtained for ABTS^●+^ and DPPH^●^ assays using non-chromans subgroup of compounds, compared to those obtained without OH-groups classification. For q^2^_LOO_ calculations, all thresholds were recalculated for each LOO model.

Assay	Simulation Type	Classification Parameter	Model (*n* = 48)
ABTS^●+^	PM6, vacuum	BDE × PA	TEAC = 0.220 + 0.809 × nOH_active_ r^2^ = 0.813, q^2^_LOO_ = 0.755
	–	–	TEAC = −0.677 + 0.803 × nOH_total_ r^2^ = 0.649, q^2^_LOO_ = 0.545
DPPH^●^	PM6, vacuum	BDE × PA	TEAC = 0.257 + 0.746 × nOH_active_ r^2^ = 0.777, q^2^_LOO_ = 0.769
	–	–	TEAC = −0.622 + 0.736 × nOH_total_ r^2^ = 0.624, q^2^_LOO_ = 0.539

**Table 4:** Models with the highest internal predictive ability obtained for ABTS^●+^ and DPPH^●^ assays using chromans subgroup of compounds, compared to those obtained without OH-group classification. For q^2^_LOO_ calculations, all thresholds were recalculated for each LOO model.

Assay	Simulation Type	Classification Parameter	Model (*n* = 40)
ABTS^●+^	AM1, COSMO	maxSD_C_ × IP	TEAC = 0.100 + 0.954 × nOH_active_ r^2^ = 0.950, q^2^_LOO_ = 0.948
	–	–	TEAC = −0.734 + 0.697 × nOH_total_ r^2^ = 0.876, q^2^_LOO_ = 0.855
DPPH^●^	AM1, COSMO	maxSD_C_ × IP	TEAC = 0.154 + 0.932 × nOH_active_ r^2^ = 0.935, q^2^_LOO_ = 0.925
	–	–	TEAC = −0.718 + 0.684 × nOH_total_ r^2^ = 0.854, q^2^_LOO_ = 0.829

## Data Availability

Not applicable.

## Supplementary Materials

The following supporting information can be downloaded at: https://www.mdpi.com/article/10.3390/molecules27072084/s1, Supplementary Tables S1 to S3: The compounds’ total OH-groups, OH-groups classified as active in radical scavenging reactions of ABTS^●^ and DPPH^●^ antiradical capacity assays by BDE, maxSD_C_, and BDE × maxSD_C_ classification descriptors and respective models’ correlation coefficients and slopes. The presented exemplary data are obtained in AM1 simulation without COSMO solvent effect corrections for complete dataset (Table S1), non-chromans subgroup (Table S2), and chromans subgroup (Table S3). Supplementary SDF files with molecular structures optimized by AM1 simulation in vacuum (AM1.vacuum.sdf) and with COSMO solvation model (AM1.cosmo.sdf). In addition to structures, OH-group positions and calculated electronic parameters (BDE, maxSD_C_, IP, PDE, PA, ETE) are provided. The electronic parameters are expressed in kcal/mol, except for maxSD_C_, which is expressed as a fraction.
