# Supplementary material for: Hybrid Classification/Regression Approach to QSAR Modeling of Stoichiometric Antiradical Capacity Assays’ Endpoints
Source: Molecules. 2022 Mar 24;27(7):2084. doi: 10.3390/molecules27072084 (PMC9000788; doi:10.3390/molecules27072084)
Supplement: Supplementary file 1 [file molecules-27-02084-s001.zip › Supplementary_Tables.pdf]

Suplementary Table S1. The compounds total OH-groups, OH-groups classified as active in radical scavenging reactions of ABTS and DPPH antiradical capacity assays by BDE, $maxSD_C$ and BDE×$maxSD_C$ and respective models correlation coefficients and slopes. The presented data are obtained for complete dataset in AM1 simulation without COSMO solvent effect corrections.

	ABTS					DPPH				
compound	nOH	BDE	$maxSD_C$	BDE×$maxSD_C$	TEAC	nOH	BDE	$maxSD_C$	BDE×$maxSD_C$	TEAC
1,5-dihydroxyanthraquinone	2	0	0	0	0.08	2	0	0	0	0.07
2,4-dihydroxybenzoic_acid	2	0	0	0	1.22	2	0	0	0	1.27
2,6-dihydroxyanthraquinone	2	0	0	0	0.07	2	0	0	0	0.08
3-hydroxyflavon	1	1	0	0	0.71	1	1	0	0	0.48
acetylshikonin	2	0	1	2	0.11	2	0	0	1	0.08
alizarin	2	1	1	1	1.07	2	1	1	1	1.05
alizarin-2-glucoside	1	0	1	1	0.11	1	0	1	1	0.10
aloe-emodin	2	0	0	0	0.08	2	0	0	0	0.04
apigenin	3	0	0	0	0.09	3	0	0	0	0.04
apigetrin	2	0	0	0	0.08	2	0	0	0	0.05
arctigenin	1	1	1	1	0.10	1	1	1	1	0.09
baicalein	3	1	1	1	2.56	3	1	1	1	2.74
baicalin	2	2	1	2	1.55	2	1	1	2	1.79
bisdemethoxycurcumin	3	2	0	0	1.18	3	2	0	0	1.02
butein	4	2	2	2	2.42	4	2	2	2	2.27
caffeic_acid	2	2	2	2	1.31	2	2	2	2	1.24
catechin	5	2	3	3	3.04	5	2	3	3	2.95
catechin_gallate	7	5	5	5	5.25	7	5	5	5	5.56
chlorogenic_acid	2	2	2	2	1.56	2	2	2	2	1.75
chrysazin	2	0	0	0	0.07	2	0	0	0	0.06
chrysin	2	0	0	0	0.08	2	0	0	0	0.05
chrysophanol	2	0	0	0	0.07	2	0	0	0	0.04
corilagin	9	9	9	9	7.76	9	9	9	9	6.98
curcumin	3	3	2	2	2.24	3	3	2	2	2.02
daidzein	2	1	0	0	0.10	2	1	0	0	0.03
daidzin	1	1	0	0	0.07	1	1	0	0	0.04
demethoxycurcumin	3	2	1	1	1.63	3	2	1	1	1.48
emodin	3	0	0	0	0.10	3	0	0	0	0.03
epicatechin	5	2	3	3	3.08	5	2	3	3	3.18
epicatechin_gallate	7	5	5	5	5.29	7	5	5	5	5.26
epigallocatechin	6	3	4	4	3.71	6	3	4	4	3.56
epigallocatechin_gallate	8	6	6	6	5.95	8	6	6	6	6.09
esculetin	2	2	2	2	2.38	2	2	2	2	2.08
esculin	1	0	1	1	0.16	1	0	1	1	0.17
ferulic_acid	1	1	1	1	1.92	1	1	0	1	1.49
galangin	3	1	1	1	1.12	3	1	1	0	0.71
gallic_acid	3	3	3	3	3.52	3	3	3	3	3.92
genistein	3	1	0	0	0.12	3	1	0	0	0.10
genistin	2	1	0	0	0.08	2	1	0	0	0.03
glycitein	2	2	1	1	0.10	2	1	1	1	0.02
hesperidin	2	1	1	1	0.10	2	1	1	1	0.08
hesperitin	3	1	1	1	0.40	3	1	1	1	0.27
isoferulic_acid	1	1	1	1	1.53	1	1	1	1	1.24
juglone	1	0	1	0	0.11	1	0	0	0	0.09
kaempferol	4	1	1	1	1.59	4	1	1	1	1.32
kaempferol-3-glucoside	3	0	1	0	0.14	3	0	1	0	0.15
luteolin	4	2	2	2	2.18	4	2	2	2	2.24
luteolin-7-glucoside	3	2	2	2	1.47	3	2	2	2	1.39
m-coumaric_acid	1	0	0	0	0.82	1	0	0	0	0.75
m-hydroxybenzoic_acid	1	0	0	0	0.03	1	0	0	0	0.07
magnolol	2	2	2	2	0.21	2	2	2	2	0.20
matairesinol	2	2	2	2	0.25	2	2	2	2	0.21
morin	5	1	2	0	2.68	5	1	1	0	2.75

naringenin	3	1	0	0	0.22	3	1	0	0	0.14
naringin	2	0	0	0	0.10	2	0	0	0	0.08
o-coumaric_acid	1	1	1	1	0.93	1	1	1	1	0.84
o-hydroxybenzoic_acid	1	0	0	0	0.04	1	0	0	0	0.05
p-coumaric_acid	1	1	0	0	1.96	1	1	0	0	1.44
p-hydroxybenzoic_acid	1	0	0	0	0.03	1	0	0	0	0.06
phloretin	4	1	0	0	1.79	4	1	0	0	1.24
physcion	2	0	0	0	0.07	2	0	0	0	0.07
piceatannol	4	2	2	2	2.53	4	2	2	2	2.35
piceatannol-3p-glucoside	3	1	1	1	1.62	3	1	1	1	1.55
procyanidin_B1	10	5	6	6	6.14	10	4	5	6	5.94
procyanidin_B2-digallate	14	10	9	10	9.18	14	10	9	10	8.79
procyanidin_C1	15	8	9	9	8.29	15	7	9	9	7.93
protocatechuic_acid	2	2	2	2	1.15	2	2	2	2	1.29
pseudopurpurin	3	2	1	2	1.62	3	2	1	1	1.70
purpurin	3	1	1	1	1.93	3	1	1	1	2.00
quercetin	5	3	3	3	4.42	5	3	3	2	4.60
quercetin-3-glucoside	4	2	3	2	2.39	4	2	3	2	2.16
quercetin-3-glucoside-7-rhamnoside	3	2	2	2	1.56	3	2	2	2	1.63
quercitrin	4	2	3	2	2.18	4	1	3	2	2.57
quinizarin	2	0	2	2	0.55	2	0	2	2	0.04
resveratrol	3	1	0	1	2.14	3	1	0	1	1.71
resveratrol-3-glucoside	2	1	0	1	1.35	2	1	0	1	1.21
resveratrol-4p-glucoside	2	0	0	0	0.56	2	0	0	0	0.62
rhein	2	0	0	0	0.08	2	0	0	0	0.06
ruberythric_acid	1	0	1	1	0.07	1	0	1	1	0.07
rutin	4	2	3	2	2.02	4	2	3	2	2.33
sappanchalcone	3	2	2	2	1.93	3	2	2	2	1.82
scopoletin	1	1	1	1	0.38	1	1	1	1	0.21
secoisolariciresinol	2	2	2	2	0.31	2	2	0	2	0.18
shikonin	2	0	1	2	0.12	2	0	1	1	0.08
syringic_acid	1	1	1	1	1.39	1	1	1	1	1.33
trolox	1	1	0	1	1.00	1	1	0	1	1.00
vanillic_acid	1	1	1	1	0.09	1	1	1	1	0.06
vitexin	3	0	1	0	0.22	3	0	1	0	0.21
r^2	0.817	0.856	0.836	0.832		0.807	0.827	0.831	0.823	
slope	0.692	0.940	0.894	0.877		0.677	0.936	0.877	0.858	

Suplementary Table S2. The compounds total OH-groups, OH-groups classified as active in radical scavenging reactions of ABTS and DPPH antiradical capacity assays by BDE, maxSD$_C$ and BDE×maxSD$_C$ and respective models correlation coefficients and slopes. The presented data are obtained for the non-chroman subgroup of the dataset in AM1 simulation without COSMO solvent effect corrections.

	ABTS					DPPH				
compound	nOH	BDE	maxSD$_C$	BDE×maxSD$_C$	TEAC	nOH	BDE	maxSD$_C$	BDE×maxSD$_C$	TEAC
1,5-dihydroxyanthraquinone	2	0	0	0	0.08	2	0	0	0	0.07
2,4-dihydroxybenzoic_acid	2	0	0	0	1.22	2	0	0	0	1.27
2,6-dihydroxyanthraquinone	2	0	0	0	0.07	2	0	0	0	0.08
acetylshikonin	2	0	1	1	0.11	2	0	0	0	0.08
alizarin	2	1	1	1	1.07	2	1	1	1	1.05
alizarin-2-glucoside	1	0	1	1	0.11	1	0	1	1	0.10
aloe-emodin	2	0	0	0	0.08	2	0	0	0	0.04
arctigenin	1	1	1	1	0.10	1	1	1	1	0.09
bisdemethoxycurcumin	3	2	0	0	1.18	3	2	0	0	1.02
butein	4	2	2	2	2.42	4	2	2	2	2.27
caffeic_acid	2	2	2	2	1.31	2	2	2	2	1.24
chlorogenic_acid	2	2	2	2	1.56	2	2	2	2	1.75
chrysazin	2	0	0	0	0.07	2	0	0	0	0.06
chrysophanol	2	0	0	0	0.07	2	0	0	0	0.04
corilagin	9	8	9	9	7.76	9	7	9	9	6.98
curcumin	3	3	2	2	2.24	3	3	2	2	2.02
demethoxycurcumin	3	2	1	1	1.63	3	2	1	1	1.48
emodin	3	0	0	0	0.10	3	0	0	0	0.03
ferulic_acid	1	1	1	1	1.92	1	1	1	1	1.49
gallic_acid	3	3	3	3	3.52	3	3	3	3	3.92
isoferulic_acid	1	1	1	1	1.53	1	1	1	1	1.24
juglone	1	0	1	0	0.11	1	0	0	0	0.09
m-coumaric_acid	1	0	0	0	0.82	1	0	0	0	0.75
m-hydroxybenzoic_acid	1	0	0	0	0.03	1	0	0	0	0.07
magnolol	2	1	2	2	0.21	2	0	2	2	0.20
matairesinol	2	2	2	2	0.25	2	2	2	2	0.21
o-coumaric_acid	1	1	1	1	0.93	1	1	1	1	0.84
o-hydroxybenzoic_acid	1	0	0	0	0.04	1	0	0	0	0.05
p-coumaric_acid	1	1	0	0	1.96	1	0	0	0	1.44
p-hydroxybenzoic_acid	1	0	0	0	0.03	1	0	0	0	0.06
phloretin	4	1	0	0	1.79	4	1	0	0	1.24
physcion	2	0	0	0	0.07	2	0	0	0	0.07
piceatannol	4	2	2	2	2.53	4	2	2	2	2.35
piceatannol-3p-glucoside	3	1	1	1	1.62	3	1	1	1	1.55
protocatechuic_acid	2	2	2	2	1.15	2	2	2	2	1.29
pseudopurpurin	3	2	1	1	1.62	3	2	1	1	1.70
purpurin	3	1	1	1	1.93	3	1	1	1	2.00
quinizarin	2	0	2	2	0.55	2	0	2	2	0.04
resveratrol	3	1	0	1	2.14	3	1	0	0	1.71
resveratrol-3-glucoside	2	1	0	1	1.35	2	1	0	0	1.21
resveratrol-4p-glucoside	2	0	0	0	0.56	2	0	0	0	0.62
rhein	2	0	0	0	0.08	2	0	0	0	0.06
ruberythric_acid	1	0	1	1	0.07	1	0	1	1	0.07
sappanchalcone	3	2	2	2	1.93	3	2	2	2	1.82
secoisolariciresinol	2	2	2	2	0.31	2	2	1	2	0.18
shikonin	2	0	1	1	0.12	2	0	1	0	0.08
syringic_acid	1	1	1	1	1.39	1	1	1	1	1.33
vanillic_acid	1	1	1	1	0.09	1	1	1	1	0.06
r^2	0.649	0.778	0.600	0.648		0.624	0.753	0.643	0.632	
slope	0.803	0.856	0.712	0.744		0.736	0.841	0.685	0.672	

Suplementary Table S3. The compounds total OH-groups, OH-groups classified as active in radical scavenging reactions of ABTS and DPPH antiradical capacity assays by BDE, $maxSD_C$ and $BDE \times maxSD_C$ and respective models correlation coefficients and slopes. The presented data are obtained for the chroman subgroup of the dataset in AM1 simulation without COSMO solvent effect corrections.

	ABTS					DPPH				
compound	nOH	BDE	$maxSD_C$	$BDE \times maxSD_C$	TEAC	nOH	BDE	$maxSD_C$	$BDE \times maxSD_C$	TEAC
3-hydroxyflavon	1	1	0	0	0.71	1	1	0	0	0.48
apigenin	3	0	0	0	0.09	3	0	0	0	0.04
apigetrin	2	0	0	0	0.08	2	0	0	0	0.05
baicalein	3	1	1	1	2.56	3	1	1	1	2.74
baicalin	2	2	1	2	1.55	2	2	1	2	1.79
catechin	5	2	3	3	3.04	5	2	3	3	2.95
catechin_gallate	7	5	5	5	5.25	7	5	5	5	5.56
chrysin	2	0	0	0	0.08	2	0	0	0	0.05
daidzein	2	1	0	0	0.10	2	1	0	0	0.03
daidzin	1	1	0	0	0.07	1	1	0	0	0.04
epicatechin	5	2	3	3	3.08	5	2	2	3	3.18
epicatechin_gallate	7	5	5	5	5.29	7	5	5	5	5.26
epigallocatechin	6	3	4	4	3.71	6	3	4	4	3.56
epigallocatechin_gallate	8	6	6	6	5.95	8	6	6	6	6.09
esculetin	2	2	2	2	2.38	2	2	2	2	2.08
esculin	1	0	1	1	0.16	1	0	1	1	0.17
galangin	3	1	1	1	1.12	3	1	1	1	0.71
genistein	3	1	0	0	0.12	3	1	0	0	0.10
genistin	2	1	0	0	0.08	2	1	0	0	0.03
glycitein	2	2	1	1	0.10	2	2	1	1	0.02
hesperidin	2	1	1	1	0.10	2	1	1	1	0.08
hesperitin	3	1	1	1	0.40	3	1	1	1	0.27
kaempferol	4	1	1	2	1.59	4	1	1	1	1.32
kaempferol-3-glucoside	3	0	1	0	0.14	3	0	0	0	0.15
luteolin	4	2	2	2	2.18	4	2	2	2	2.24
luteolin-7-glucoside	3	2	2	2	1.47	3	2	2	2	1.39
morin	5	1	1	2	2.68	5	1	1	1	2.75
naringenin	3	1	0	0	0.22	3	1	0	0	0.14
naringin	2	0	0	0	0.10	2	0	0	0	0.08
procyanidin_B1	10	6	6	6	6.14	10	4	5	6	5.94
procyanidin_B2-digallate	14	10	9	10	9.18	14	10	9	10	8.79
procyanidin_C1	15	8	9	9	8.29	15	8	9	9	7.93
quercetin	5	3	3	3	4.42	5	3	3	3	4.60
quercetin-3-glucoside	4	2	3	2	2.39	4	2	3	2	2.16
quercetin-3-glucoside-7-rhamnoside	3	2	2	2	1.56	3	2	2	2	1.63
quercitrin	4	2	3	2	2.18	4	2	3	2	2.57
rutin	4	2	3	2	2.02	4	2	3	2	2.33
scopoletin	1	1	1	1	0.38	1	1	1	1	0.21
trolox	1	1	0	1	1.00	1	1	0	1	1.00
vitexin	3	0	1	0	0.22	3	0	1	0	0.21
r^2	0.876	0.900	0.921	0.950		0.854	0.865	0.903	0.925	
slope	0.697	1.002	0.965	0.954		0.684	1.008	0.961	0.931	
